# Supplementary material for: Long-chain ceramides are cell non-autonomous signals linking lipotoxicity to endoplasmic reticulum stress in skeletal muscle
Source: Nat Commun. 2022 Apr 1;13:1748. doi: 10.1038/s41467-022-29363-9 (PMC8975934; doi:10.1038/s41467-022-29363-9)
Supplement: Supplementary file 1 — Supplementary Information [file 41467_2022_29363_MOESM1_ESM.pdf]

Supplementary Information

Long chain ceramides are cell non-autonomous signals linking lipotoxicity to endoplasmic reticulum stress in skeletal muscle

Ben D McNally[1], Dean F Ashley[1], Lea Hänschke[2], Hélène N Daou[3]†, Nicole T Watt[3]†, Steven A Murfitt[1], Amanda DV MacCannell[3], Anna Whitehead[3], T. Scott Bowen[4], Francis WB Sanders[1], Michele Vacca[1,5], Klaus K Witte[3], Graeme R Davies[6], Reinhard Bauer[2], Julian L Griffin[1, 7], Lee D Roberts[3*]

1. Department of Biochemistry, University of Cambridge, Cambridge, CB2 1GA, UK.
2. Life & Medical Sciences Institute (LIMES) Development, Genetics & Molecular Physiology Unit, University of Bonn, Carl-Troll-Straße, 31 53115 Bonn, Germany
3. School of Medicine, University of Leeds, Leeds, LS2 9JT, UK.
4. Faculty of Biological Sciences, University of Leeds, Leeds, LS2 9JT, UK.
5. Clinica Medica "Frugoni", Interdisciplinar Department of Medicine, University of Bari "Aldo Moro", Italy
6. Bioscience Metabolism, Research and Early Development, Cardiovascular, Renal and Metabolism, BioPharmaceuticals R&D, AstraZeneca, Cambridge, UK
7. Department of Metabolism, Digestion and Reproduction, Imperial College London, London, U.K.

† Equal Contribution

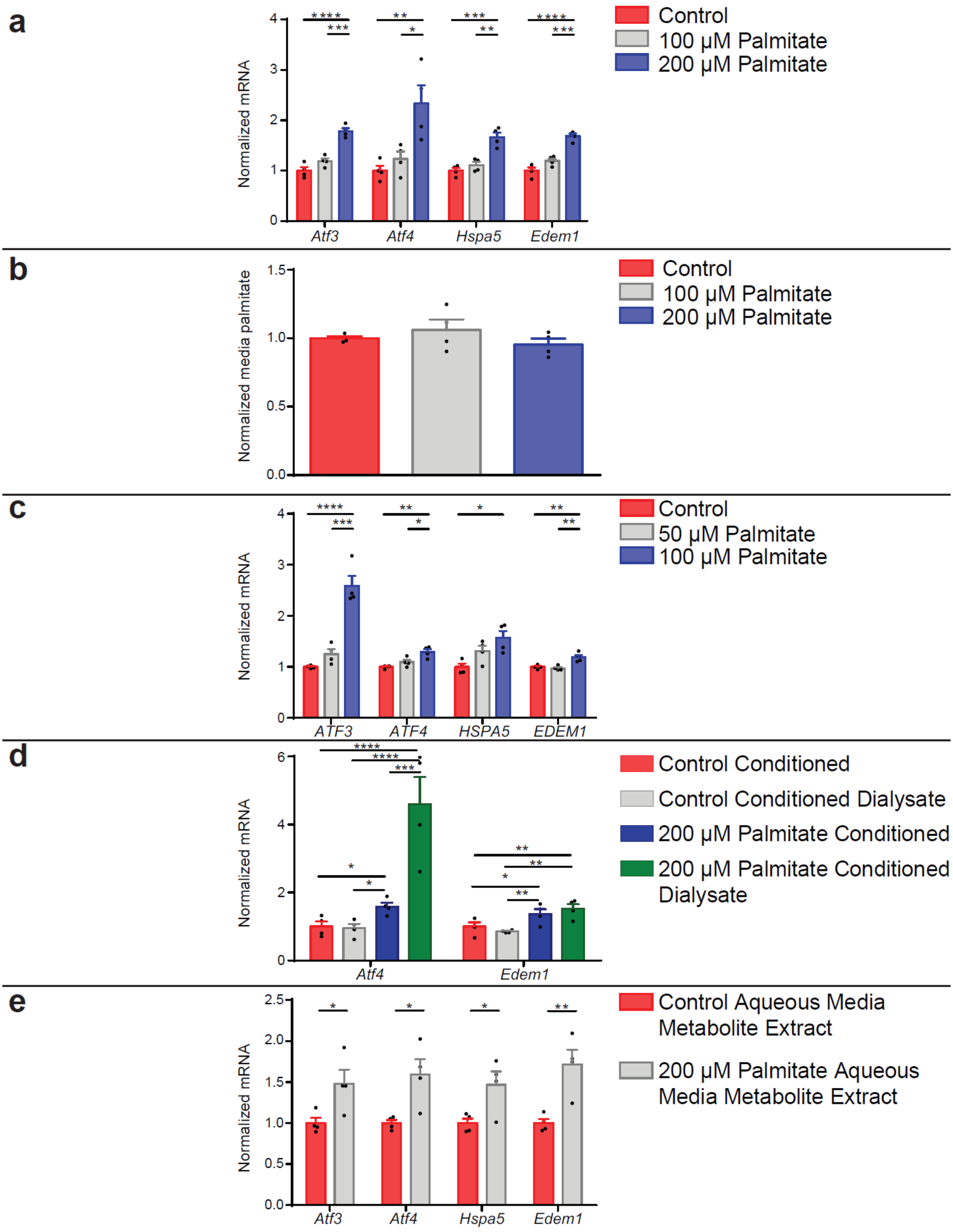

Supplementary Fig. 1 a. Myotubes secrete a cell non-autonomous unfolded protein response (UPR) -inducing lipid signal in response to chronic palmitate-induced lipotoxicity. Activating transcription factor 3 (*Atf3*), activating transcription factor 4 (*Atf4*),

Heat Shock Protein Family A (Hsp70) Member 5 (*Hspa5*) and ER Degradation Enhancing Alpha-Mannosidase Like Protein 1 (*Edem1*) unfolded protein response (UPR) gene expression in C2C12 myotubes treated with 100 µM (grey) and 200 µM palmitate (blue) or fatty acid free bovine serum albumin (BSA) (red) vehicle for 6 days (n = 4; one-way ANOVA; control vs 200 µM palmitate, *Atf3 P* < 0.0001, *Atf4 P* = 0.0073, *Hspa5 P* = 0.0003, *Edem1 P* < 0.0001; 100 µM palmitate vs 200 µM palmitate, *Atf3 P* = 0.0002, *Atf4 P* = 0.02, *Hspa5 P* = 0.001, *Edem1 P* = 0.0003). **b**. Free palmitate concentrations in conditioned media collected from control C2C12 myotubes (red) and C2C12 myotubes treated with 100 µM (grey) and 200 µM palmitate (blue) (n = 4). **c**. *ATF3, ATF4, HSPA5* and *EDEM1* UPR gene expression in HSkMCs treated with 50 (grey) and 100 µM palmitate (blue) or BSA control (red) (n = 4; one-way ANOVA; control vs 200 µM palmitate, *ATF3 P < 0.0001* , *ATF4 P* = 0.002, *Hspa5 P* = 0.012, *Edem1 P* = 0.0065; 100 µM palmitate vs 200 µM palmitate, *ATF3 P* = 0.0001, *ATF4 P* = 0.02, *Edem1 P* = 0.003). **d.** UPR gene expression in C2C12 myotubes receiving 1 kDa molecular weight cut off dried and reconstituted dialysate from conditioned media collected from C2C12 myotubes treated with 200 µM palmitate or BSA vehicle control (control conditioned = red, control conditioned dialysate = grey, palmitate conditioned = blue, palmitate conditioned dialysate = green) (n = 4, one-way ANOVA; control conditioned vs 200 µM palmitate conditioned, *Atf4 P* = 0.02, *Edem1 P* = 0.049; control conditioned vs 200 µM palmitate conditioned dialysate, *Atf4 P* < 0.0001, *Edem1 P* = 0.009; control conditioned dialysate vs 200 µM palmitate conditioned, *Atf4 P* = 0.011, *Edem1 P* = 0.0097; control conditioned dialysate vs 200 µM palmitate conditioned dialysate, *Atf4 P* < 0.0001, *Edem1 P* = 0.0018). **e**. UPR gene expression in myotubes receiving reconstituted aqueous extract isolated from conditioned media collected from C2C12 myotubes treated with 200 µM palmitate (grey) or BSA vehicle control (red) (n = 4; two tailed Student's T-test; *Atf3 P* = 0.04, *Atf4 P* = 0.02, *Hspa5 P* = 0.03, *Edem1 P* = 0.008).*

$P \leq 0.05$, ** $P < 0.01$, *** $P < 0.001$, **** $P < 0.0001$. Data are expressed as mean ± SEM with individual data points shown. Source data are provided as a Source Data file.

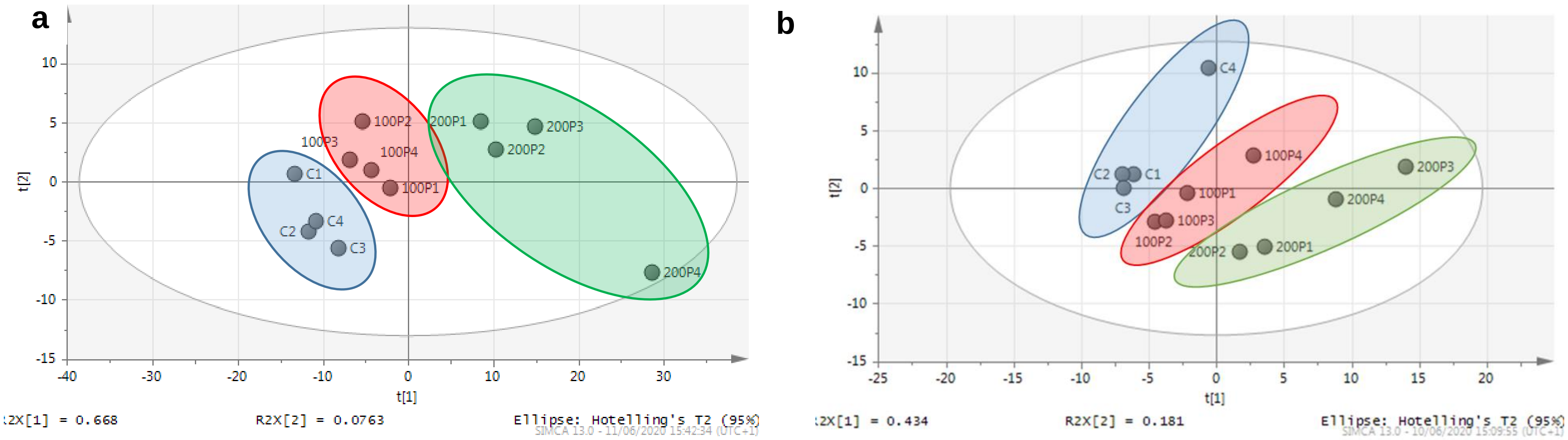

Supplementary Figure 2. Multivariate analysis of Liquid Chromatography – Mass Spectrometry (LC-MS) lipidomic data from conditioned media collected from C2C12 myotubes treated with 100 µM and 200 µM palmitate or fatty acid free bovine serum albumin control. **a**. Partial least Squares – discriminant analysis (PLS-DA) plot of positive mode LC-MS data showing separation of media from control (blue), 100 µM palmitate treated (red) and 200 µM palmitate treated (green) C2C12 myocytes ($Q^2 = 0.668$). **b**. PLS-DA plot of negative mode LC-MS data showing separation of media from control (blue), 100 µM palmitate treated (red) and 200 µM palmitate treated (green) C2C12 myocytes ($Q^2 = 0.434$). (n = 4). Source data are provided as a Source Data file.

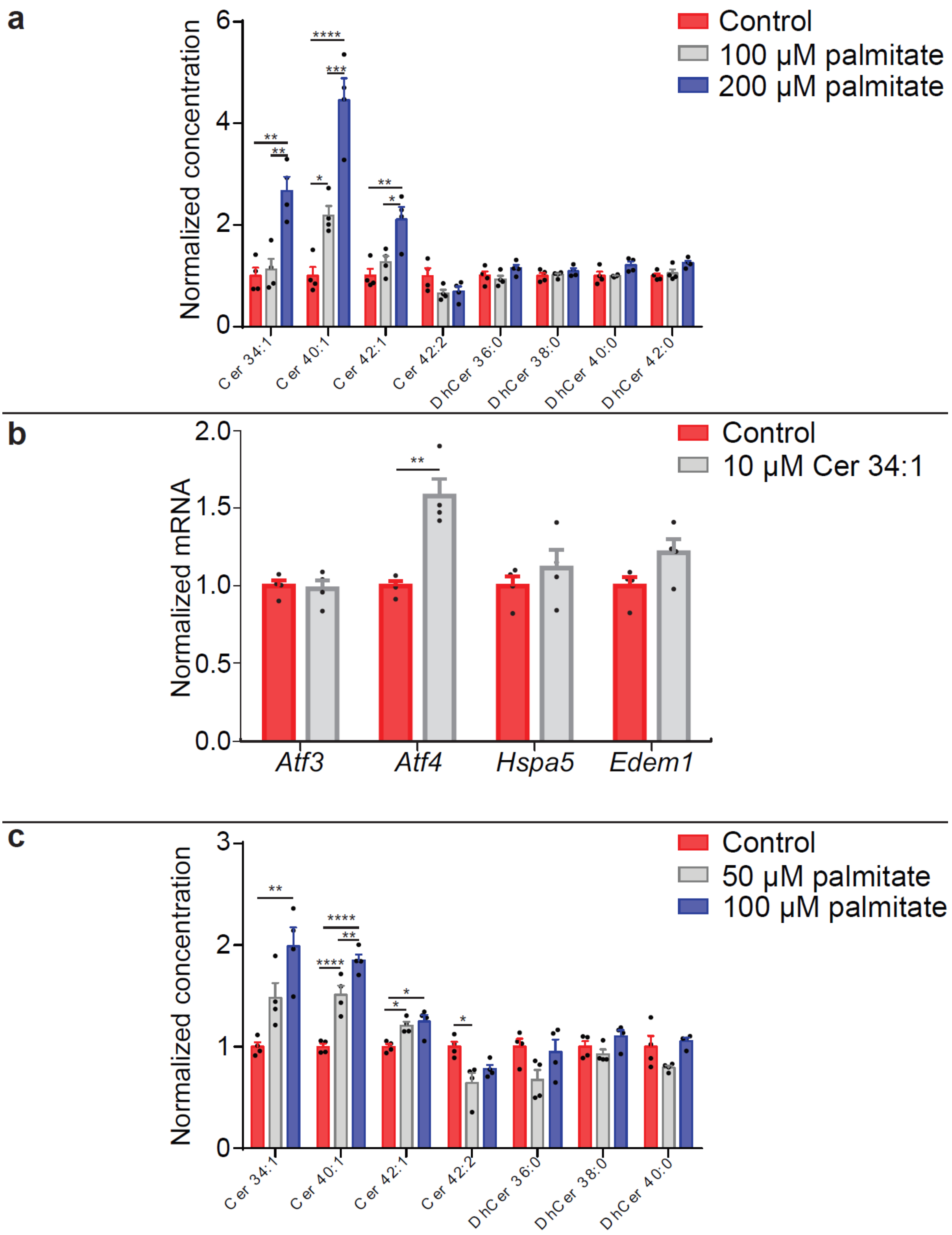

Supplementary Figure 3. a. Lipotoxicity induced long-chain ceramide secretion from myotubes. Ceramide (Cer) and Dihydroceramide (DhCer) lipid species profile detected by liquid chromatography – mass spectrometry (LC-MS) lipidomics in conditioned media collected from C2C12 myotubes treated with vehicle control (red) or 100 μM (grey) and 200

μM palmitate (blue) (n = 4; One-Way ANOVA; control vs 200 µM palmitate, Cer 34:1 P = 0.0012, Cer 40:1 P < 0.0001, Cer 42:1 P = 0.004; control vs 100 µM palmitate, Cer 40:1 P = 0.043; 100 µM palmitate vs 200 µM palmitate Cer 34:1 P = 0.002, Cer 40:1 P = 0.001, Cer 42:1 P = 0.02). **b.** Activating transcription factor 3 (*Atf3*), activating transcription factor 4 (*Atf4*), Heat Shock Protein Family A (Hsp70) Member 5 (*Hspa5*) and ER Degradation Enhancing Alpha-Mannosidase Like Protein 1 (*Edem1*) unfolded protein response gene expression in C2C12 myotubes treated with vehicle control (red) or 10 µM ceramide (Cer) 34:1 (grey) (n = 4; two-tailed Student's T-test; *Atf 4* P = 0.002). **c.** Cer and DhCer lipid species profile detected by LC-MS lipidomics in conditioned media collected from human primary skeletal myotubes treated with vehicle control (red) or 50 µM (grey) and 100 µM (blue) palmitate. (n = 4; one-way ANOVA; control vs 50 µM palmitate Cer 40:1 P = 0.001, Cer 42:1 P = 0.025, Cer 42:2 P = 0.012; control vs 100 µM palmitate, Cer 34:1 P = 0.0017, Cer 40:1 P < 0.0001, Cer 42:1 P = 0.01; 50 µM palmitate vs 100 µM palmitate Cer 40:1 P = 0.014). * P < 0.05, ** P < 0.01, *** P < 0.001, **** P < 0.0001. Data are expressed as mean ± SEM with individual data points shown. Source data are provided as a Source Data file.

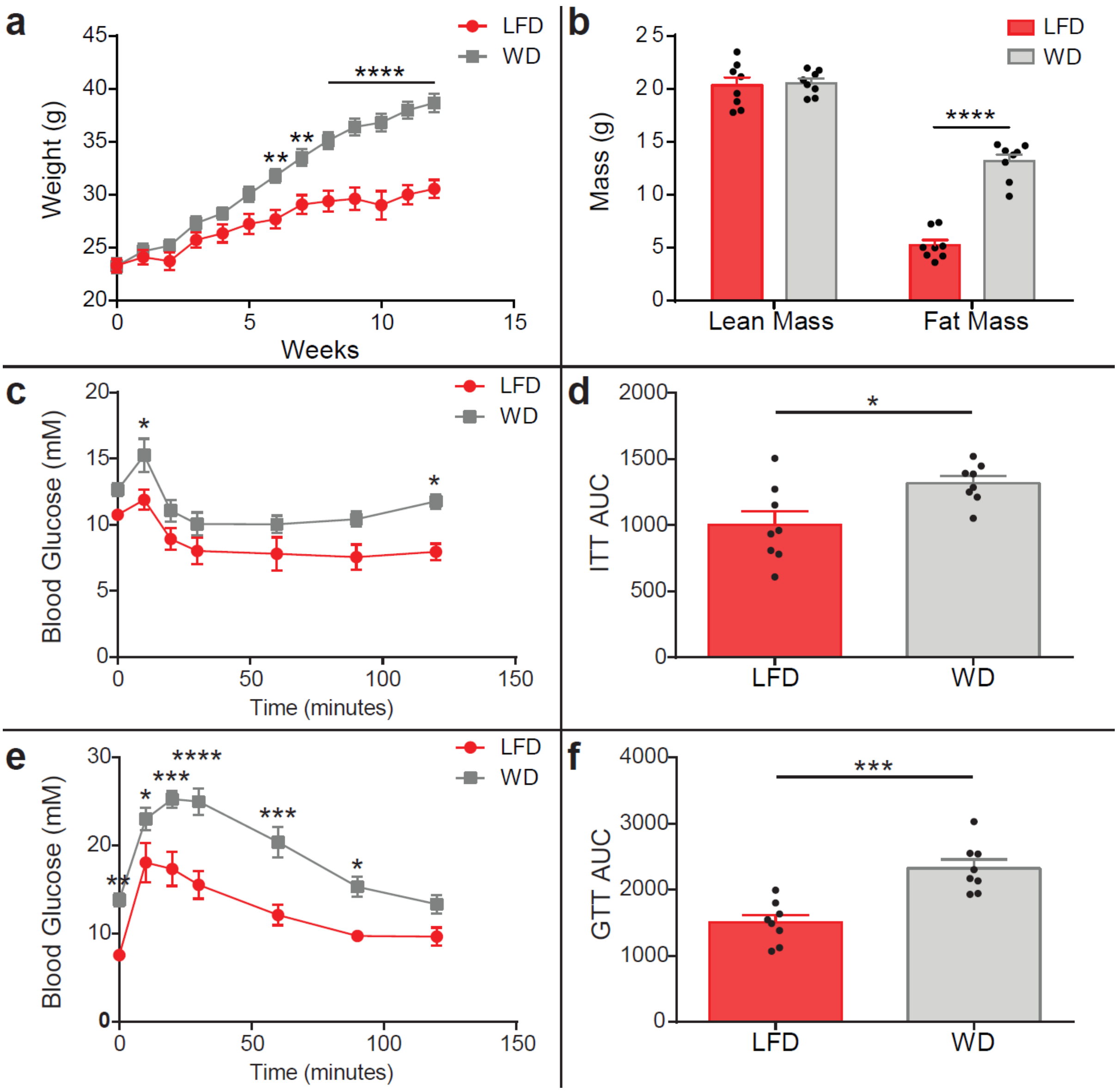

Supplementary Figure 4. Long-chain ceramides are enriched in murine and human skeletal muscle during metabolic disease a. Body weight of C57Bl6 mice fed either a low-fat control diet (LFD) or a western diet (WD) for 12 weeks (n = 8; two-way ANOVA; week 6 $P = 0.0041$, week 7 $P = 0.0016$, week 8 – 12 $P < 0.0001$). **b**. Lean mass and fat mass of LFD and WD fed mice (n = 8; two-tailed Student's T-test; Lean Mass $P < 0.0001$). **c**. Insulin tolerance test (ITT) (n = 8; two-way ANOVA; 10 mins $P = 0.028$, 120 mins $P = 0.01$) and **d**. ITT area under the curve (AUC) (n = 8; two-tailed Student's T-test; $P = 0.017$) of mice fed an LFD and WD. ITT conducted at week 11 of the study. **e**. Glucose tolerance test (GTT) (n = 8; two-way ANOVA; 0 min $P = 0.0051$, 10 min $P = 0.02$, 20 min $P = 0.0003$, 30 min $P < 0.0001$,

60 min *P* = 0.0002, 90 min *P* = 0.012) and **f**. GTT AUC (n = 8; two-tailed Student's T-test; *P* = 0.0003) of mice fed an LFD and WD conducted at week 10 of the study. * $P \leq 0.05$, ** $P < 0.01$, *** $P < 0.001$, **** $P < 0.0001$. Data are expressed as mean ± SEM. LFD = red, WD = grey. Source data are provided as a Source Data file.

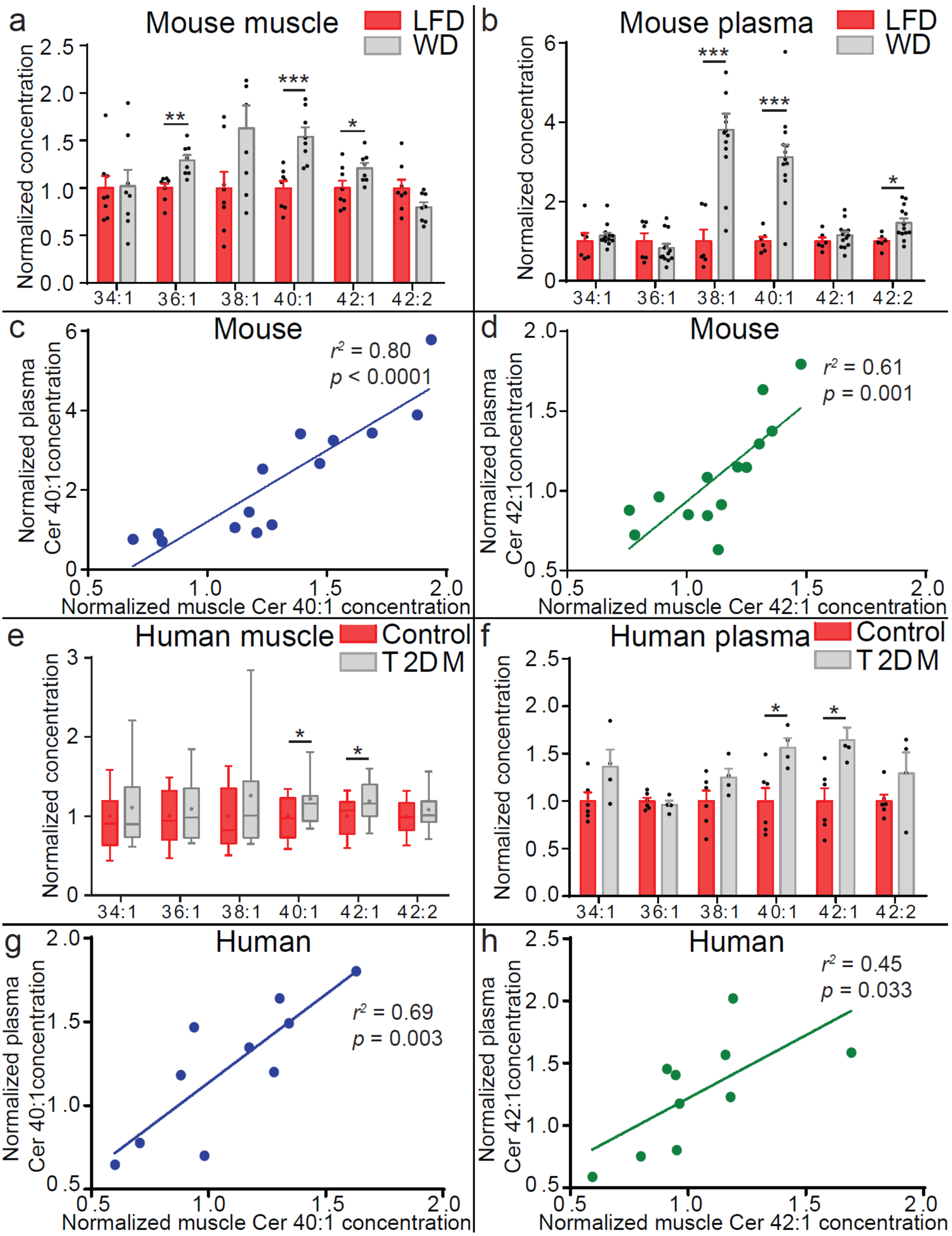

Supplementary Figure 5. Plasma and muscle long-chain ceramide concentrations are increased in metabolic disease in mouse and humans. C57Bl6 mice were fed either a low-fat control diet (LFD) or a western diet (WD) for 12 weeks. **a**. Ceramide profiles in soleus

muscle of LFD (red) and WD (grey) fed mice analysed by liquid chromatography - mass spectrometry (LC-MS) (n = 8; two-tailed Student's T-test; Cer 36:1 $P = 0.0014$, Cer 40:1 $P = 0.00056$, Cer 42:1 $P = 0.045$). **b**. Ceramide profiles in plasma of LFD (red) and WD (grey) fed mice measured by LC-MS (LFD n = 6, WD n = 13; two-tailed Student's T-test; Cer 38:1 $P = 0.00037$, Cer 40:1 $P = 0.00031$, Cer 42:1 $P = 0.017$). **c**. The correlation of muscle to plasma Cer 40:1 (blue; n = 14, two-tailed Pearson correlation; $r^2 = 0.8$, $P < 0.0001$) concentrations in LFD and WD mice. **d**. The correlation of muscle to plasma Cer 42:1 (green; n = 14, two-tailed Pearson correlation; $r^2 = 0.61$, $P = 0.001$) concentrations in LFD and WD mice. **e**. Ceramide profiles measured by LC-MS in skeletal muscle biopsies taken from patients with type 2 diabetes (T2D) (grey) and controls (red) (control n = 52, T2D n = 21; two-tailed Student's T-test; Cer 40:1 $P = 0.033$, Cer 42:1 $P = 0.017$). **f.** Ceramide profiles measured by LC-MS in blood plasma taken from patients with T2D (grey) and controls (red) (control n = 6, T2D n = 4; two-tailed Student's T-test; Cer 40:1 $P = 0.018$, Cer 42:1 $P = 0.012$). **g.** The correlation of skeletal muscle to plasma Cer 40:1 (blue; n = 10; two-tailed Pearson correlation; $r^2 = 0.69$, $P = 0.003$) concentrations in patients with T2D and controls. **h.** The correlation of skeletal muscle to plasma Cer 42:1 (green; n = 10; two-tailed Pearson correlation; $r^2 = 0.45$, $P = 0.033$) concentrations in patients with T2D and controls. * $P \leq 0.05$, ** $P < 0.01$, *** $P < 0.001$, **** $P < 0.0001$. Data in bar charts are expressed as mean ± SEM with individual data points shown. Box and whisker plots show 25th to 75th percentile (box) min to max (whiskers), mean (+) and median (−). Source data are provided as a Source Data file.

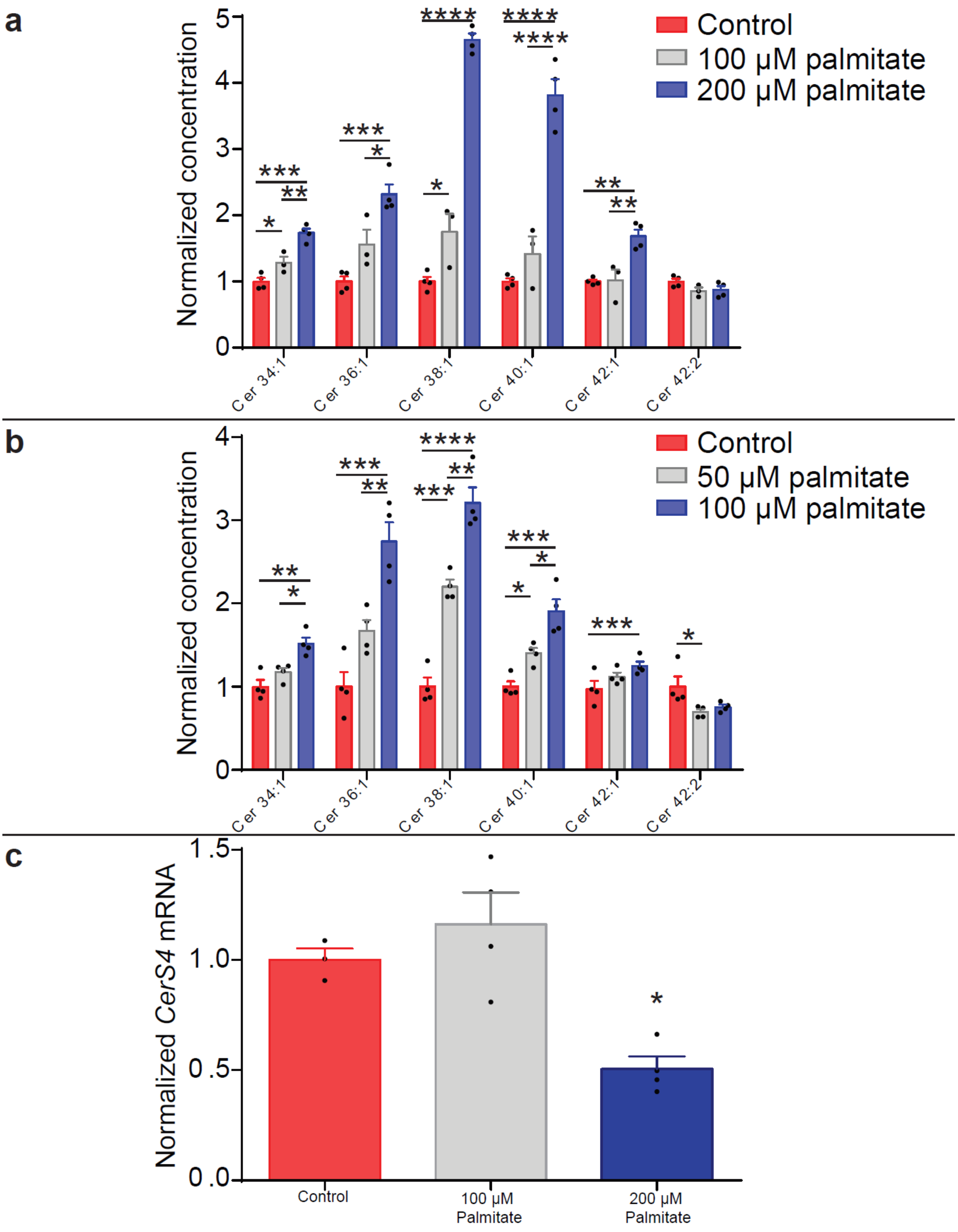

Supplementary Figure 6. Palmitate induces long-chain ceramide synthesis through the *de novo* pathway. a. Intracellular ceramide (Cer) lipid species profile detected by liquid chromatography – mass spectrometry lipidomics in C2C12 myotubes treated with vehicle control (red) 100 µM (grey) and 200 µM (blue) palmitate (n = 4 control, 200 µM palmitate; n

= 3 100 µM palmitate; One-Way ANOVA; control vs 200 µM palmitate, Cer 34:1 *P* = 0.0001, Cer 36:1 *P* = 0.0005, Cer 38:1 *P* < 0.0001, Cer 40:1 *P* < 0.0001, Cer 42:1 *P* = 0.0026; control vs 100 µM palmitate, Cer 34:1 *P* = 0.05, Cer 38:1 *P* = 0.018; 100 µM palmitate vs 200 µM palmitate Cer 34:1 *P* = 0.005, Cer 36:1 *P* = 0,02, Cer 38:1 *P* < 0.0001, Cer 40:1 *P* < 0.0001, Cer 42:1 *P* = 0.0047) **b.** Intracellular ceramide (Cer) lipid species profile detected by liquid chromatography – mass spectrometry lipidomics in primary human skeletal myotubes treated with vehicle control (red) 50 µM (grey) and 100 µM (blue) palmitate (n = 4; one-Way ANOVA; control vs 200 µM palmitate, Cer 34:1 *P* = 0.0014, Cer 36:1 *P* = 0.0002, Cer 38:1 *P* < 0.0001, Cer 40:1 *P* = 0.0003, Cer 42:1 *P* = 0.05; control vs 100 µM palmitate, Cer 38:1 *P* = 0.0003, Cer 40:1 *P* = 0.043, Cer 42:2 *P* = 0.043; 100 µM palmitate vs 200 µM palmitate Cer 34:1 *P* = 0.0018, Cer 36:1 *P* = 0.0063, Cer 38:1 *P* = 0.0012, Cer 40:1 *P* = 0.014). **c.** *CerS4* expression in C2C12 myotubes treated with either 100 µM (grey) or 200 µM palmitate (blue) or the BSA vehicle control (red) (control n = 3; 100 µM and 200 µM palmitate n = 4; one-way ANOVA; control vs 200 µM palmitate *P* = 0.025). * *P* ≤ 0.05, ** *P* < 0.01, *** *P* < 0.001, **** *P* < 0.0001. Data are expressed as mean ± SEM with individual data points shown. Source data are provided as a Source Data file.

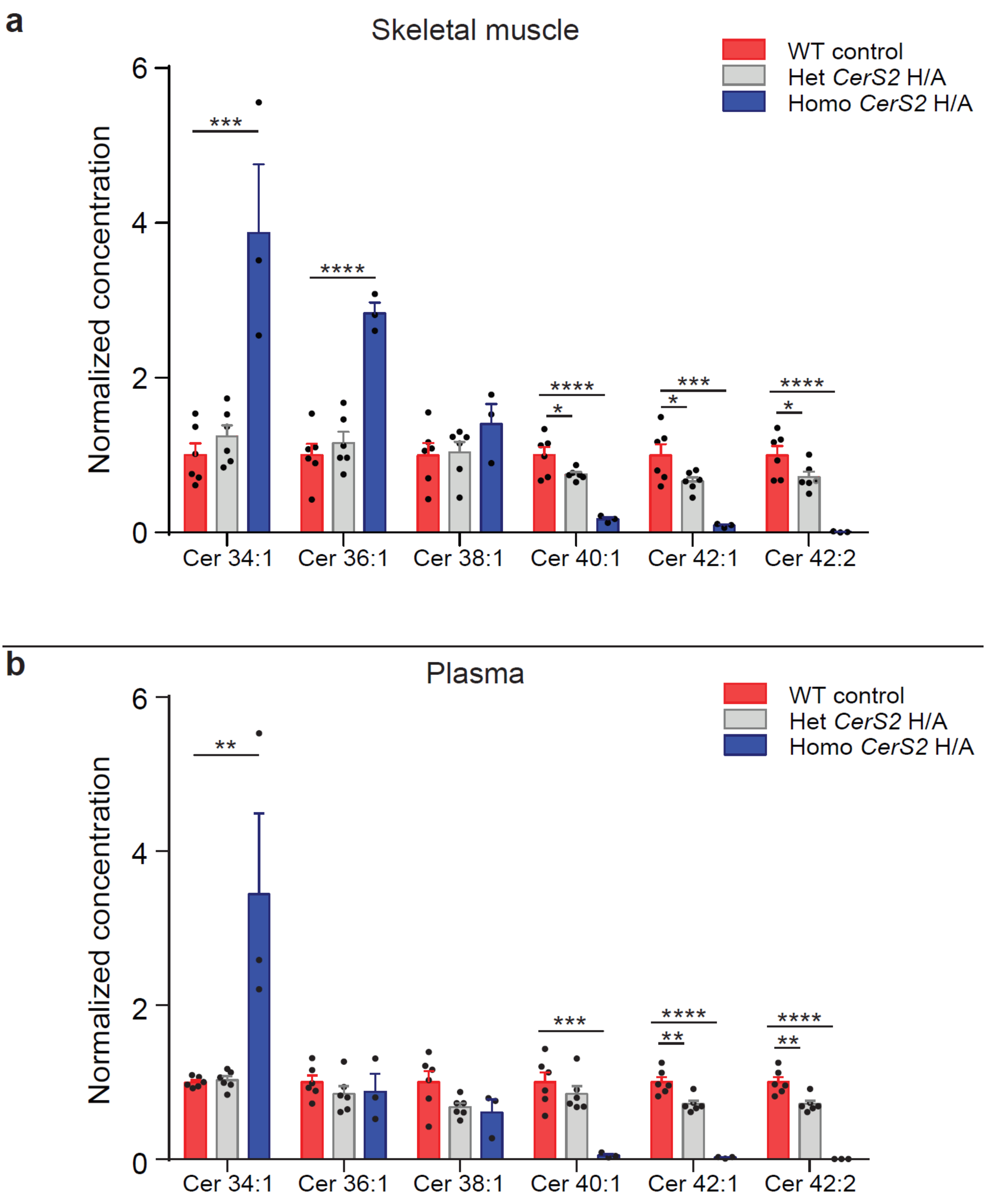

Supplementary Figure 7. The ceramide profile of heterozygous and homozygous *CerS2* H/A transgenic mice. a. Ceramide lipid profile of the soleus muscle of transgenic mice heterozygous (Het *CerS2* H/A; grey) and homozygous (Homo *CerS2* H/A; blue) for catalytically inactivated ceramide synthase 2 compared to wildtype (WT; red) littermate

controls (WT, Het *CerS2* H/A n = 6; Homo *CerS2* H/A n = 3; one-way ANOVA; WT vs Het *CerS2* H/A, Cer 40:1 $P = 0.03$, Cer 42:1 $P = 0.03$, Cer 42:2 $P = 0.042$; WT vs Homo *CerS2* H/A, Cer 34:1 $P = 0.0003$, Cer 36:1 $P < 0.0001$, Cer 40:1 $P < 0.0001$, Cer 42:1 $P = 0.0005$, Cer 42:2 $P < 0.0001$). **b.** Ceramide lipid profile of the blood plasma of Het *CerS2* H/A (grey) and Homo *CerS2* H/A (blue) compared to WT (red) controls (WT, Het *CerS2* H/A n = 6; Homo *CerS2* H/A n = 3; one-way ANOVA; WT vs Het *CerS2* H/A, Cer 42:1 $P = 0.0018$, Cer 42:2 $P = 0.0018$; WT vs Homo *CerS2* H/A, Cer 34:1 $P = 0.0018$, Cer 40:1 $P = 0.0006$, Cer 42:1 $P < 0.0001$, Cer 42:2 $P < 0.0001$). * $P \leq 0.05$, ** $P < 0.01$, *** $P < 0.001$, **** $P < 0.0001$. Data are expressed as mean ± SEM with individual data points shown. Source data are provided as a Source Data file.

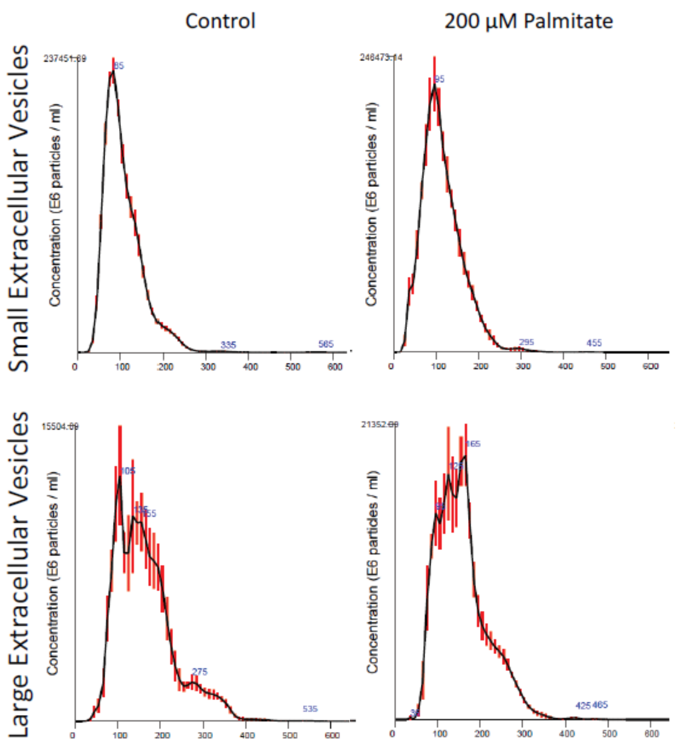

Supplementary Figure 8. NanoSight analysis showing size distributions of small extracellular vesicle (exosome) and large extracellular vesicle (microvesicle) populations in conditioned media harvested from C2C12 myotubes treated with 200 uM palmitate**.** Source data are provided as a Source Data file.

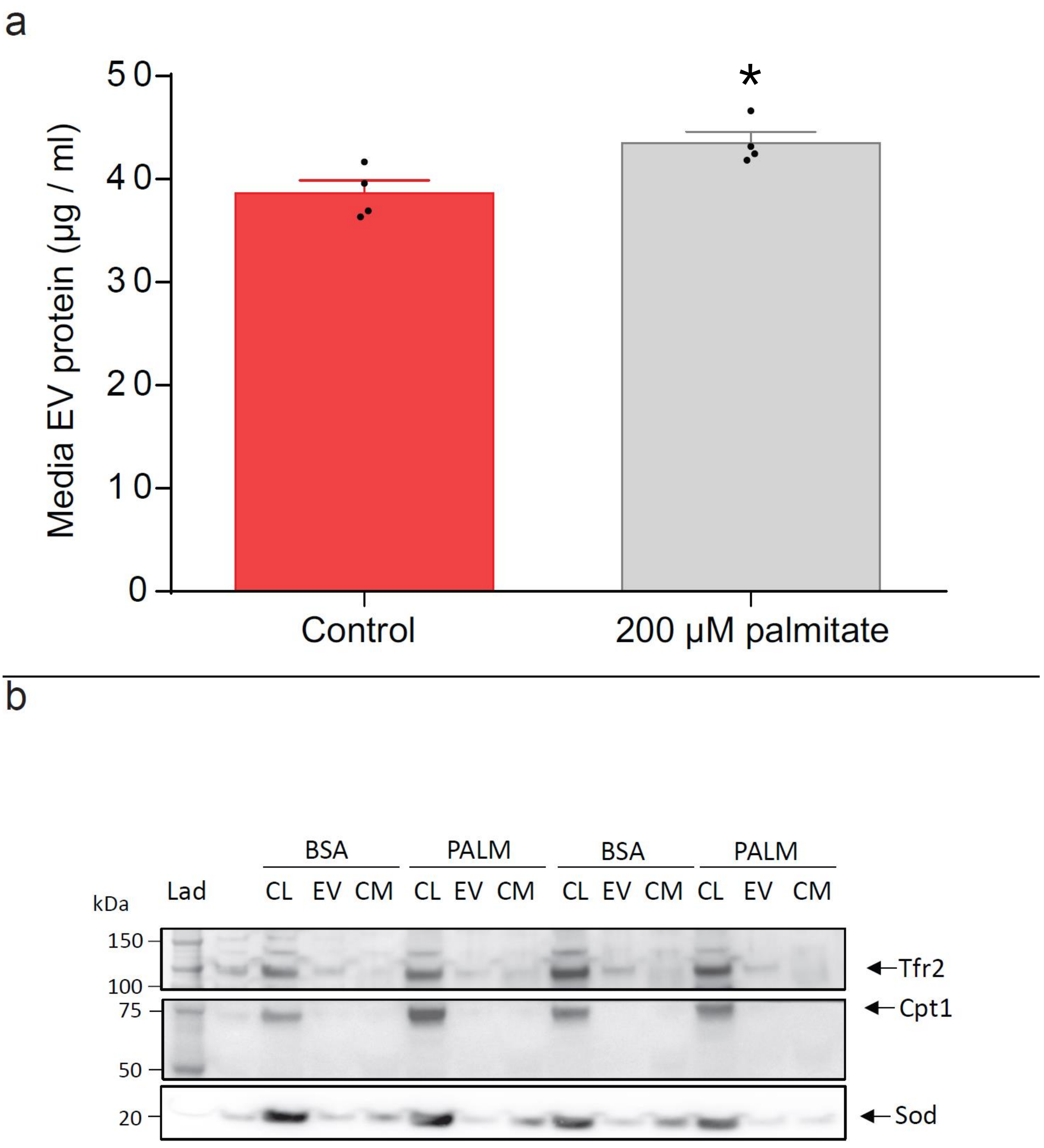

Supplementary Figure 9. Extracellular vesicle characterisation. a. The protein quantification of media extracellular vesicle (EV) isolations from conditioned media of BSA control (red) and 200 µM palmitate-treated (grey) C2C12 myotubes (n = 4; two-tailed Student's T-Test; P = 0.024) **b.** Immunoblotting of the plasma membrane associated transmembrane

protein, transferrin receptor 2 (Tfr2), the cytosolic protein superoxide dismutase 1 (Sod) recovered in EVs, and the mitochondrial protein carnitine palmitoyltransferase 1 (Cpt1), a component of the non-EV co-isolated structure in EV isolations (EV), conditioned media (CM) and C2C12 myocyte whole cell lysates (CL) from BSA vehicle control and 200 µM palmitate-treated C2C12 myotubes. Molecular weight ladder (Lad). * $P \leq 0.05$. Data in bar graphs are expressed as mean ± SEM with individual data points shown. Source data are provided as a Source Data file.

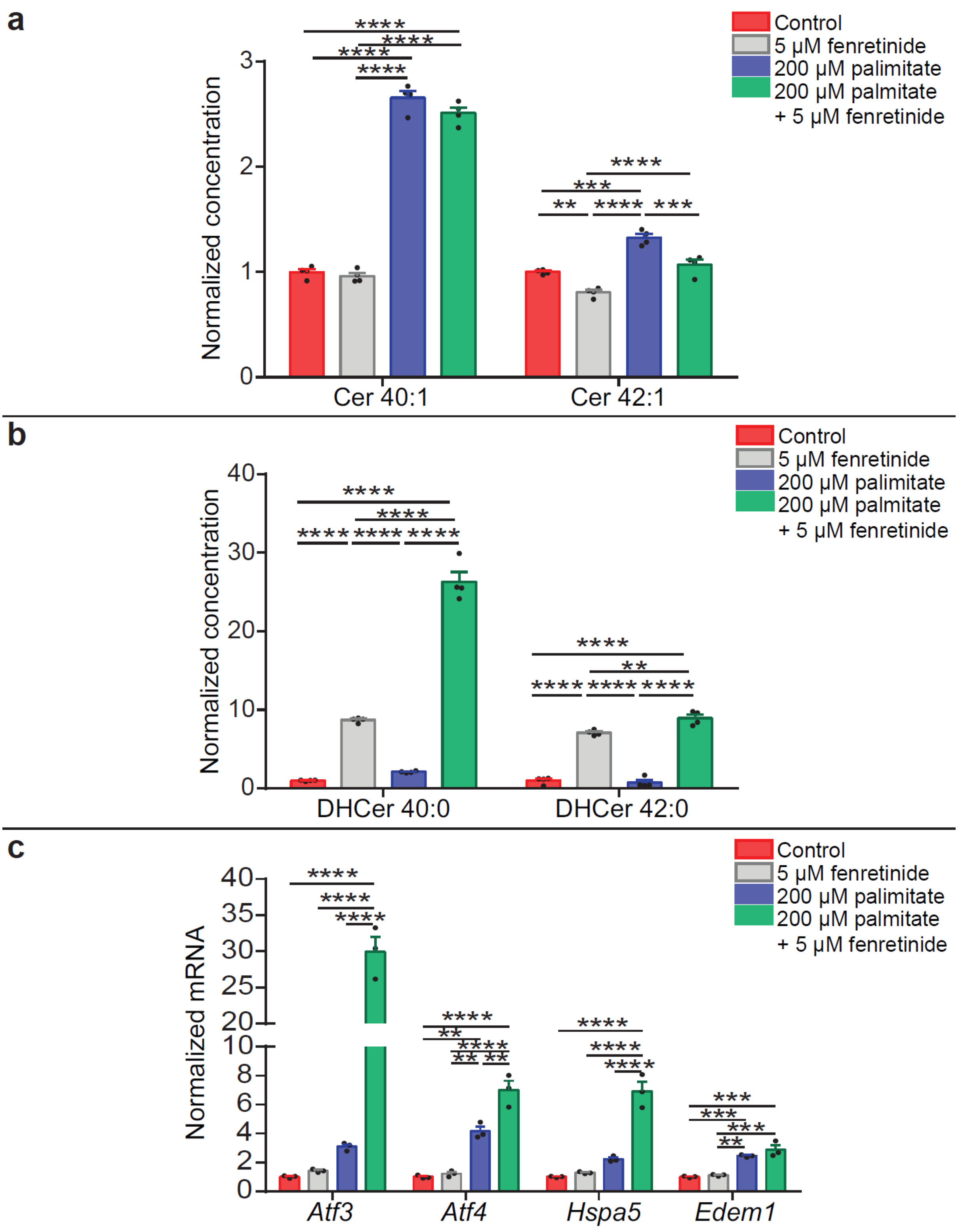

Supplementary Fig 10. Ceramide-induced accumulation of dihydroceramides increases UPR gene expression. a. Intracellular ceramides Cer 40:1 and Cer 42:1 in C2C12 myotubes treated with 200 µM palmitate or BSA, in conjunction with 5µM fenretinide (n = 4; one-way ANOVA; control vs 5 µM fenretinide, Cer 42:1 P = 0.002; control vs 200 µM palmitate, Cer

40:1 *P* < 0.0001, Cer 42:1 *P* < 0.0001; control vs 200 µM palmitate + 5 µM fenretinide, Cer 40:1 *P* < 0.0001; 200 µM palmitate + 5 µM fenretinide, Cer 40:1 *P* < 0.0001, Cer 42:1 *P* < 0.0001; 5 µM fenretinide vs 200 µM palmitate + 5 µM fenretinide, Cer 40:1 *P* < 0.0001, Cer 42:1 *P* =0.0004, 200 µM palmitate vs 200 µM palmitate + 5 µM fenretinide, Cer 42:1 *P* = 0.0004). **b**. Intracellular dihydroceramides DHCer 40:0 and DHCer 42:0 in C2C12 myotubes treated with 200 µM palmitate or BSA, in conjunction with 5µM fenretinide (n = 4; one-way ANOVA; control vs 5 µM fenretinide, DHCer 40:0 *P* < 0.0001, DHCer 42:0 *P* < 0.0001; control vs 200 µM palmitate + 5 µM fenretinide, DHCer 40:0 *P* < 0.0001, DHCer 42:0 *P* < 0.0001; 5 µM fenretinide vs 200 µM palmitate, DHCer 40:0 *P* < 0.0001, DHCer 42:0 *P* < 0.0001; 5 µM fenretinide vs 200 µM palmitate + 5 µM fenretinide, DHCer 40:0 *P* < 0.0001, DHCer 42:0 *P* = 0.002; 200 µM palmitate vs 200 µM palmitate + 5 µM fenretinide, DHCer 40:0 *P* < 0.0001, DHCer 42:0 *P* < 0.0001). **c**. Activating transcription factor 3 (*Atf3*), activating transcription factor 4 (*Atf4*), Heat Shock Protein Family A (Hsp70) Member 5 (*Hspa5*) and ER Degradation Enhancing Alpha-Mannosidase Like Protein 1 (*Edem1*) UPR gene expression in C2C12 myotubes treated with palmitate or BSA, in conjunction with 5µM Fenretinide (n = 3; one-way ANOVA; control vs 200 µM palmitate, *Atf4 P* = 0.001, *Edem1 P* = 0.0008; control vs 200 µM palmitate + 5 µM fenretinide, *Atf3 P* < 0.0001, *Atf4 P* < 0.0001, *Hspa5 P* < 0.0001, *Edem1 P* = 0.0002; 5 µM fenretinide vs 200 µM palmitate, *Atf4* P = 0.0014, *Edem1 P* =0.001; 5 µM fenretinide vs 200 µM palmitate + 5 µM fenretinide, *Atf3 P* < 0.0001, *Atf4 P* < 0.0001, *Hspa5 P* < 0.0001, *Edem1 P* = 0.0002; 200 µM palmitate vs 200 µM palmitate + 5 µM fenretinide, *Atf3 P* < 0.0001, *Atf4 P* = 0.0014, *Hspa5 P* < 0.0001). * *P* ≤ 0.05, ** *P* < 0.01, *** *P* < 0.001, **** *P* < 0.0001. Data are expressed as mean ± SEM with individual data points shown. Control = red; 5 µM fenretinide = grey; 200 µM palmitate = blue; 200 µM palmitate + 5 µM fenretinide = green. Source data are provided as a Source Data file.

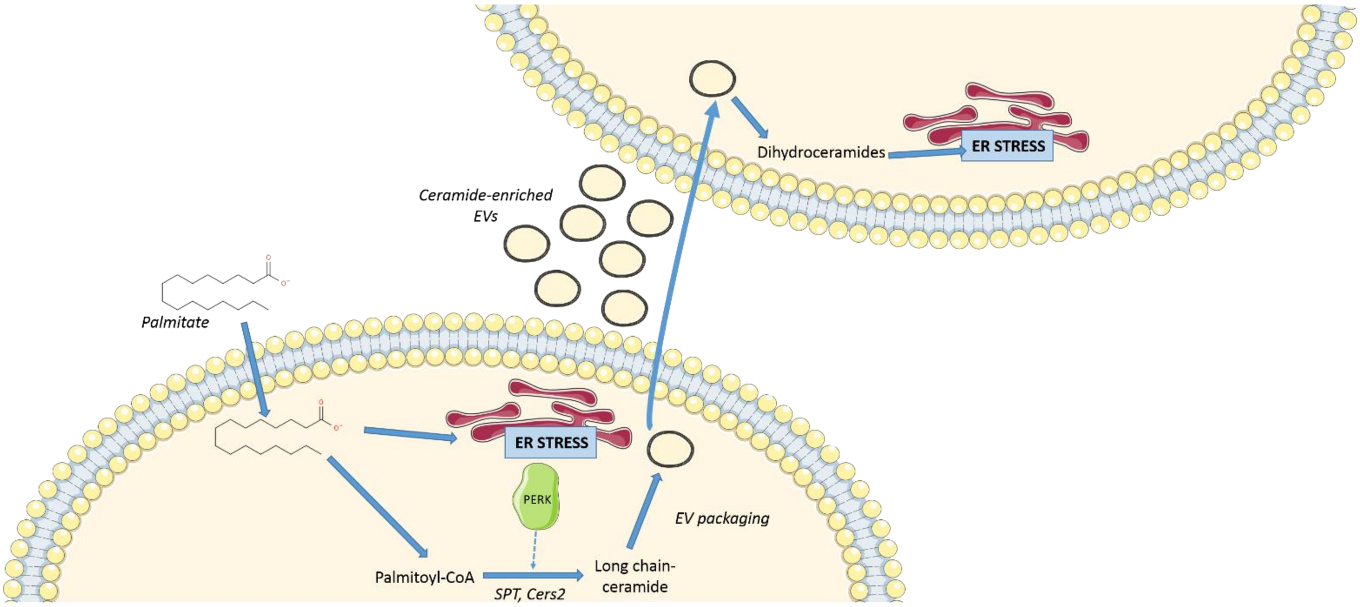

Supplementary Figure 11. Long chain ceramides are cell non-autonomous signals that propagate the activation of endoplasmic reticulum (ER) stress. Chronic palmitate treatment increases the synthesis of long-chain ceramide 40:1 and 42:1 via the *de novo* synthesis pathway and CerS2 in a Perk-dependent manner. These ceramides are released from the cell in extracellular vesicles (EVs) and can activate expression of unfolded protein response (UPR) genes in neighbouring cells. This induction of UPR signalling may be as a result of dihydroceramide accumulation.

Supplementary Table 1. Morphological parameters for human patient volunteers. Data is mean +/- SEM. Statistical analysis of dichotomous variables was by χ^2. Continuous variables were analysed by two-tailed Student's t-test.

	Control patients (n = 53)	Patients with Type 2 Diabetes (n = 22)	*P* - value
Male, n	43 (81 %)	19 (86 %)	0.59
Age (years)	72.05 ± 11.54	73.04 ± 10.01	0.73
Weight (kg)	80.6 ± 13.83	93.64 ± 22.50	0.0025
BMI (kg/m^2)	27.43 ± 4.829	30.94 ± 7.129	0.016
HbA1c (mmol/mol)	39.73	56.81	$3.67e^{-8}$

Supplementary Table 2 PCR primer details giving species, gene, commercial vendor, vendor catalogue number and primer reference position in NCBI reference sequence.

Species	Gene	Commercial Vendor	Catalogue Number	Primer Reference Position
Human	*ATF3*	Qiagen (GeneGlobe)	PPH00408C	1617 (NM_001674)
	ATF4		PPH02016A	1242 (NM_001675)
	HSPA5		PPH00158E	3889 (NM_005347)
	EDEM1		PPH12935B	2113 (NM_014674)
	EIF2AK3		PPH10874A	3583 (NM_004836)
	CERS2		PPH16029F	599 (NM_022075)
	DEGS1		PPH16415A	247 (NM_003676)
Mouse	*Atf3*		PPM04669C	402 (NM_007498)
	Atf4		PPM04670E	1484 (NM_009716)
	Hspa5		PPM03586B	1573 (NM_022310)
	Edem1		PPM26189B	1687 (NM_138677)
	Cers2		PPM32482A	1022 (NM_029789)
	Cers4		PPM28333A	1238 (NM_026058)
