# Supplementary material for: Long-chain ceramides are cell non-autonomous signals linking lipotoxicity to endoplasmic reticulum stress in skeletal muscle
Source: Nat Commun. 2022 Apr 1;13:1748. doi: 10.1038/s41467-022-29363-9 (PMC8975934; doi:10.1038/s41467-022-29363-9)
Supplement: Supplementary file 2 — Reporting Summary [file 41467_2022_29363_MOESM2_ESM.pdf]

nature portfolio

Corresponding author(s): Dr Lee D Roberts

Last updated by author(s): Mar 1, 2022

Reporting Summary

Nature Portfolio wishes to improve the reproducibility of the work that we publish. This form provides structure for consistency and transparency in reporting. For further information on Nature Portfolio policies, see our Editorial Policies and the Editorial Policy Checklist.

Statistics

For all statistical analyses, confirm that the following items are present in the figure legend, table legend, main text, or Methods section.

n/a	Confirmed	
☐	☒	The exact sample size (*n*) for each experimental group/condition, given as a discrete number and unit of measurement
☐	☒	A statement on whether measurements were taken from distinct samples or whether the same sample was measured repeatedly
☐	☒	The statistical test(s) used AND whether they are one- or two-sided<br>*Only common tests should be described solely by name; describe more complex techniques in the Methods section.*
☐	☒	A description of all covariates tested
☐	☒	A description of any assumptions or corrections, such as tests of normality and adjustment for multiple comparisons
☐	☒	A full description of the statistical parameters including central tendency (e.g. means) or other basic estimates (e.g. regression coefficient) AND variation (e.g. standard deviation) or associated estimates of uncertainty (e.g. confidence intervals)
☐	☒	For null hypothesis testing, the test statistic (e.g. *F*, *t*, *r*) with confidence intervals, effect sizes, degrees of freedom and *P* value noted<br>*Give P values as exact values whenever suitable.*
☒	☐	For Bayesian analysis, information on the choice of priors and Markov chain Monte Carlo settings
☒	☐	For hierarchical and complex designs, identification of the appropriate level for tests and full reporting of outcomes
☐	☒	Estimates of effect sizes (e.g. Cohen's *d*, Pearson's *r*), indicating how they were calculated

Our web collection on statistics for biologists contains articles on many of the points above.

Software and code

Policy information about availability of computer code

Data collection	Mass spectrometry lipidomic data was collected in Xcalibur (Thermo Scientific, version 2.2). RT-qPCR data was collected using StepOne Software (version 2.1 Applied Biosystems). Extracellular vesicle NanoSight data was collected in NTA3.0 software (NanoSight, Malvern Panalytical). Confocal microscope data was collected in ZEN software (Version 2.60, ZEISS). Western blot data was imaged/collected using GeneSys version 1.8.3 (Syngene).
Data analysis	Multivariate data analysis was performed using SIMCA-P+ 13.0 (Umetrics AB, Umeå, Sweden). Univariate data analysis was performed using Prism Graphpad (version 6.02). Xcalibur .raw files were converted to .mzML files using MS Convert (Version 3, Proteowizard). Mass spectrometry lipidomic data was analyzed using XCMS (Version 3.4, Scripps Institute). RT-qPCR data was analyzed using StepOne Software (version 2.1 Applied Biosystems). Western blot densitometry analysis was performed using GeneSys version 1.8.3 (Syngene). Extracellular vesicle population data analysis was performed in NTA3.0 software (NanoSight, Malvern Panalytical)

For manuscripts utilizing custom algorithms or software that are central to the research but not yet described in published literature, software must be made available to editors and reviewers. We strongly encourage code deposition in a community repository (e.g. GitHub). See the Nature Portfolio guidelines for submitting code & software for further information.

Data

Policy information about availability of data

All manuscripts must include a data availability statement. This statement should provide the following information, where applicable:

- Accession codes, unique identifiers, or web links for publicly available datasets
- A description of any restrictions on data availability
- For clinical datasets or third party data, please ensure that the statement adheres to our policy

Source data are provided with this paper. The data generated in this study are provided in the Source Data file. The Lipid Maps database was used in lipid identification from mass spectrometry data (https://www.lipidmaps.org/).

Field-specific reporting

Please select the one below that is the best fit for your research. If you are not sure, read the appropriate sections before making your selection.

☒ Life sciences ☐ Behavioural & social sciences ☐ Ecological, evolutionary & environmental sciences

For a reference copy of the document with all sections, see nature.com/documents/nr-reporting-summary-flat.pdf

Life sciences study design

All studies must disclose on these points even when the disclosure is negative.

Sample size	Sample sizes for experiments (primary outcomes: change in glucose tolerance, circulating, tissue, cell media ceramide concentrations; tissue and cell Unfolded Protein Response gene expression) were calculated using power calculations. Standard deviations were based on previous studies or on the available literature. Power calculations were made using beta = 0.8 and alpha = 0.05.
Data exclusions	Data was only excluded where whole experiments failed (based on positive or negative controls, animals or cells failed to meet experiment endpoint or variance of internal standards) (this is relevant to experiments presented in Fig 2c, Fig 4f, Fig 4i), or where limited material (e.g. human tissues) were exhausted (experiments presented in Fig 3c&d).
Replication	Replicate experiments were successful. All experiments were replicated a minimum of three times with the exception of the human volunteer and animal studies (which were sufficiently powered), which were each performed independently once due to ethical limitations on repeated human and animal studies.
Randomization	All samples were randomly assigned to experimental groups. Animals were weight matched and randomly assigned to experimental groups. Cells in culture wells were randomly assigned to study groups.
Blinding	For animal studies researchers were blinded to experimental grouping by core technicians in the animal facility to avoid bias and aid reproducibility. Experimentalists were blinded to study groups for cell culture endpoint data collection and analysis. Experimentalists were blinded to human patient data and group allocation for human tissue analysis by clinical and research staff.

Reporting for specific materials, systems and methods

We require information from authors about some types of materials, experimental systems and methods used in many studies. Here, indicate whether each material, system or method listed is relevant to your study. If you are not sure if a list item applies to your research, read the appropriate section before selecting a response.

Materials & experimental systems

n/a	Involved in the study	
☐	☒	Antibodies
☐	☒	Eukaryotic cell lines
☒	☐	Palaeontology and archaeology
☐	☒	Animals and other organisms
☐	☒	Human research participants
☒	☐	Clinical data
☒	☐	Dual use research of concern

Methods

n/a	Involved in the study	
☒	☐	ChIP-seq
☒	☐	Flow cytometry
☒	☐	MRI-based neuroimaging

Antibodies

Antibodies used	Primary antibodies: TrfR (H68.4) Product # 13-6800, lot VH307351 1:1000 dilution, GAPDH (1D4) catalogue #MA1-16757 lot# WH3346651A, 1:1000 dilution – Invitrogen, Paisley UK), CPT1a (EPR21843-71-2F) (catalogue # ab234111 ABCAM, lot. GR3254231-8) 1:1000 dilution, SOD (71G8) mAb #4266 lot 2 1:1000 dilution, XBP-1s (E9V3E) mAb #40435 lot# 1 1:1000 dilution, CHOP (L63F7) mAb

	#2895 lot # 13 1:1000 dilution (All Cell Signalling Technologies, London UK). Peroxidase-conjugated secondary antibodies (sheep anti-mouse (NA931V, 1:3000 dilution, GE Healthcare, Amersham, UK) and goat anti-rabbit (G21234, 1:3000 dilution, Invitrogen, Paisley UK).
Validation	Primary Antibodies<br>Transferrin Receptor Antibody (H68.4; Invitrogen):<br>Antibody specificity was demonstrated by siRNA mediated knockdown of target protein. HeLa cells were transfected with Transferrin Receptor siRNA and loss of signal was observed in Western Blot using Anti-Transferrin Receptor monoclonal Antibody (Product # 13-6800) (https://www.thermofisher.com/antibody/product/Transferrin-Receptor-Antibody-clone-H68-4-Monoclonal/13-6800).<br><br>GAPDH Monoclonal Antibody (1D4) MA1-16757 (Invitrogen):<br>Antibody specificity was demonstrated by siRNA mediated knockdown of target protein. HeLa cells were transfected with GAPDH siRNA and loss of signal was observed in Western Blot using Anti-GAPDH monoclonal Antibody. https://www.thermofisher.com/antibody/product/GAPDH-Antibody-clone-1D4-Monoclonal/MA1-16757<br><br>Recombinant Anti-CPT1A antibody [EPR21843-71-2F] (ab234111): Suitable for: Flow Cyt (Intra), WB, IHC-P. Knockout validated. https://www.abcam.com/cpt1a-antibody-epr21843-71-2f-ab234111.html. This antibody has been validated using SimpleChIP® Enzymatic Chromatin IP Kits.<br><br>SOD1 (71G8) mAb #4266: Suitable for: Flow Cyt (Intra), WB, IHC-P<br>https://www.cellsignal.de/products/primary-antibodies/sod1-71g8-mouse-mab/4266?site-search-type=Products&N=4294956287&Ntt=anti-mouse+sod1&fromPage=plp. This antibody has been validated using SimpleChIP® Enzymatic Chromatin IP Kits.<br><br>XBP-1s (E9V3E) mAb #40435: Validated for Western Blotting, Immunofluorescence (Immunocytochemistry), Flow Cytometry, Chromatin IP. https://www.cellsignal.co.uk/products/primary-antibodies/xbp-1s-e9v3e-rabbit-mab/40435. This antibody has been validated using SimpleChIP® Enzymatic Chromatin IP Kits.<br><br>CHOP (L63F7) Mouse mAb #2895. https://www.cellsignal.co.uk/products/primary-antibodies/chop-l63f7-mouse-mab/2895 This antibody has been validated using SimpleChIP® Enzymatic Chromatin IP Kits. Validated for Western Blotting, Immunoprecipitation, Immunofluorescence (Immunocytochemistry), Flow Cytometry, Chromatin IP.<br><br>Secondary Antibodies<br>Goat anti-Rabbit IgG (H+L), HRP (G-21234, Invitrogen). Validated as a secondary antibody for westernblot, IHC, IP and ELISA. https://www.thermofisher.com/antibody/product/Goat-anti-Rabbit-IgG-H-L-Cross-Adsorbed-Secondary-Antibody-Polyclonal/G-21234<br><br>Sheep anti-mouse IgG, peroxidase-linked species-specific whole antibody (NA931). Validated for westerblot using Hybond ECL membrane containing tubulin protein and detected with primary monoclonal anti-tubulin and secondary antibody NA931 anti-mouse IgG. cytivialifesciences.com/en/us/shop/protein-analysis/blotting-and-detection/blotting-standards-and-reagents/amersham-ecl-hrp-conjugated-antibodies-p-06260#related-documents

Eukaryotic cell lines

Policy information about cell lines

Cell line source(s)	C2C12 cells were purchased from the European Collection of Authenticated Cell Culture Operated by Public Health England through Sigma Aldrich (Cat no. 91031101). Adult primary human skeletal myoblasts were purchased from Cell Applications Inc (Cat no. 150-05a).
Authentication	Human primary skeletal myoblasts (Cell Application Inc Cat no. 150-05a) are authenticated by the supplier. In addition myoblasts are tested for their ability to attach, spread, proliferate and differentiate in culture conditions. The cells are authenticated for their ability to form multinucleated myotubes using morphological and gene/protein expression markers following differentiation.<br><br>The C2C12 cell line was purchased from Sigma Aldrich (Cat no. 91031101) and authenticated by the providers. The cells are authenticated for their ability to form multinucleated myotubes using morphological and gene expression markers for Myod1, Tnni1 and Tnni2 using RT-qPCR (RT2 primer assays, Qiagen; Myod1 - PPM04481A-200, Tnni1 - PPM29072A-200, Tnni2-PPM28627E-200) following differentiation.
Mycoplasma contamination	All cells have been tested for mycoplasma contamination by the providers (negative results).
Commonly misidentified lines<br>(See ICLAC register)	No commonly misidentified cells lines were used in this study.

Animals and other organisms

Policy information about studies involving animals; ARRIVE guidelines recommended for reporting animal research

Laboratory animals	For western diet studies five-week-old male C57Bl6j mice were purchased from Harlan Laboratory Ltd. Animals were housed with a 12hr light/dark cycle at 20+/-4 degrees Celsius and 40 - 60 % humidity.<br>For CerS2 H/A mouse studies, eight week old male heterozygous, homozygous and wild type littermates were used. Animals were

	housed with a 12hr light/dark cycle at room temperature and 40 - 60 % humidity.
Wild animals	The study did not involve wild animals.
Field-collected samples	The study did not involve animals collected from the field.
Ethics oversight	Animal studies were regulated under the UK Animals (Scientific Procedures) Act 1986, following ethical review by the University of Cambridge Animal Welfare and Ethical Review Body (AWERB). All procedures were carried out in accordance with U.K. Home Office protocols by a personal license holder under a Home Office Project Licence. Animal studies were also conducted in accordance and under authority of the University of Bonn local ethics committee and following German national authority mandates.

Note that full information on the approval of the study protocol must also be provided in the manuscript.

Human research participants

Policy information about studies involving human research participants

Population characteristics	Seventy five patients were recruited for this study. The primary outcome was mass spectrometry analysis of lipids in skeletal muscle (pectoralis major) biopsies. Inclusion criteria for subjects included:<br>Normal cardiac function on echocardiography<br>No symptoms or past diagnosis of heart failure<br>Able and willing to give informed consent<br><br>Exclusion criteria for subjects included:<br>Unable or unwilling to give consent<br>Symptoms possibly due to heart failure<br>Current or previous diagnosis of cancer, inflammatory or musculoskeletal disease (e.g. rheumatoid arthritis)<br>Ongoing infection or sepsis, type 1 diabetes mellitus, heart failure.<br><br>Eligible patients were grouped into two age and sex-matched cohorts based on the presence (n = 22) or absence (n = 53) of type 2 Diabetes Mellitus (T2DM). The T2DM cohort had a previous diagnosis of T2DM (>3 months) and/or was receiving treatment for diabetes. In such cases, T2DM was defined as a documented history of diabetes, fasting plasma glucose ≥ 7.0 mmol/L, plasma glucose ≥ 11.1 mmol/L two hours after the OGTT or a glycated haemogolobin (HbA1c) ≥ 6.5% (≥ 48 mmol/L).<br><br>The recruited patient demographics are given in Supplementary Table 1. For the control cohort, age = 72.05 ± 11.54 (years), Sex = 81% Male (43/53), weight = 80.6 ± 13.83 (Kg), Body Mass Index = 27.43 ± 4.829, Hba1c = 39.73 mmol/mol. Data is Mean +/- SEM.<br><br>For the T2DM cohort, age = 73.04 ± 10.01 (years), Sex = 86% (19/22), weight = 93.64 ± 22.50 (Kg), Body Mass Index = 30.94 ± 7.129, Hba1c = 56.81 mmol/mol. Data is Mean +/- SEM.
Recruitment	Eligible and consecutive patients undergoing routine de-novo pacemaker implantation at Leeds General Infirmary, Leeds Teaching Hospital Trust, UK volunteered to participate in the study and provided written consent. Self-selection bias is unlikely to be an important factor. All patients fulfilling the inclusion criteria are approached consecutively during the clinical list. Given the nature of the sampling process, which is pain free, and therefore goes unnoticed during the routine clinical procedure, patients rarely decline to participate. In the time frame of the recruitment to this study we had one patient decline to participate.
Ethics oversight	The study is approved by the Leeds West Research Ethics Committee (11/YH/0291) and Leeds Teaching Hospitals Trust R&D committee (CD11/10015) and conforms to the Declaration of Helsinki.

Note that full information on the approval of the study protocol must also be provided in the manuscript.
